# Nanoscale Chemical Analysis of Thin Film Solar Cell Interfaces Using Tip-Enhanced Raman Spectroscopy

**DOI:** 10.1021/acsami.3c17115

**Published:** 2024-03-18

**Authors:** Siiri Bienz, Giulia Spaggiari, Davide Calestani, Giovanna Trevisi, Danilo Bersani, Renato Zenobi, Naresh Kumar

**Affiliations:** † Department of Chemistry and Applied Biosciences, ETH Zurich, Vladimir-Prelog-Weg 1-5/10, 8093 Zurich, Switzerland; ‡ Department of Mathematical, Physical and Computer Sciences, University of Parma, Parco Area delle Scienze 7/A, I-43124 Parma, Italy; § Institute of Materials for Electronics and Magnetism, National Research Council, Parco Area delle Scienze 37/A, I-43124 Parma, Italy

**Keywords:** Sb_2_Se_3_ solar cell, tip-enhanced Raman spectroscopy, thin film solar cells, nanoscale imaging, interfacial analysis

## Abstract

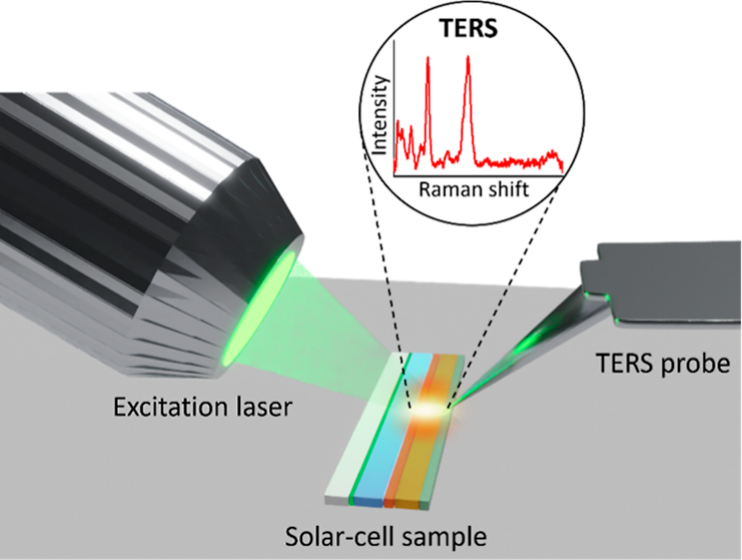

Interfacial regions play a key role in determining the overall power conversion efficiency of thin film solar cells. However, the nanoscale investigation of thin film interfaces using conventional analytical tools is challenging due to a lack of required sensitivity and spatial resolution. Here, we surmount these obstacles using tip-enhanced Raman spectroscopy (TERS) and apply it to investigate the absorber (Sb_2_Se_3_) and buffer (CdS) layers interface in a Sb_2_Se_3_-based thin film solar cell. Hyperspectral TERS imaging with 10 nm spatial resolution reveals that the investigated interface between the absorber and buffer layers is far from uniform, as TERS analysis detects an intermixing of chemical compounds instead of a sharp demarcation between the CdS and Sb_2_Se_3_ layers. Intriguingly, this interface, comprising both Sb_2_Se_3_ and CdS compounds, exhibits an unexpectedly large thickness of 295 ± 70 nm attributable to the roughness of the Sb_2_Se_3_ layer. Furthermore, TERS measurements provide compelling evidence of CdS penetration into the Sb_2_Se_3_ layer, likely resulting from unwanted reactions on the absorber surface during chemical bath deposition. Notably, the coexistence of ZnO, which serves as the uppermost conducting layer, and CdS within the Sb_2_Se_3_-rich region has been experimentally confirmed for the first time. This study underscores TERS as a promising nanoscale technique to investigate thin film inorganic solar cell interfaces, offering novel insights into intricate interface structures and compound intermixing.

## Introduction

In the realm of thin film solar cells, Sb_2_Se_3_-based cells have gathered significant attention over the past decade^1^ due to their nontoxic nature, greater abundance compared to other conventional absorber materials, band gap of 1.0–1.2 eV,^2^ and a high absorption coefficient.^3^ Notably, their highest reported power conversion efficiency stands at 10%.^4^ However, despite the substantial interest and promising performance of Sb_2_Se_3_ thin film solar cells, their efficiency is still significantly lower than the theoretical prediction because of several unresolved questions.^5^ Particularly, the understanding of chemistry occurring at the film interfaces, which is pivotal for overall performance, remains limited. This limited understanding primarily results from the nanoscale dimensions of these interfaces that pose a challenge for the conventional analytical tools. Specifically, the interface between the absorber and buffer layer exerts a substantial influence on the thin film solar cell’s performance and efficiency. To enhance the power conversion efficiency, it is imperative to prevent obstacles to charge carrier transport, recombination losses, and the formation of defects that can act as traps at the absorber–buffer interface.

Previous investigations of thin film solar cells, such as Cu(In,Ga)Se_2_ solar cells, relied on techniques like energy-dispersive X-ray spectroscopy,^6,7^ X-ray photoelectron spectroscopy,^8,9^ X-ray emission spectroscopy,^9^ or Auger electron spectroscopy.^10^ However, most of these methods offer a limited spatial resolution, demand a vacuum environment, and provide only elemental information. On the other hand, electron microscopy methods like scanning electron microscopy (SEM) and transmission electron microscopy can offer nanoscale resolution and have been employed to visualize solar cell cross sections, grain sizes, and the surface roughness of individual layers.^7,8,11^ Nonetheless, despite their widespread use, these techniques do not yield structural information of complex mixtures at the nanoscale. Other frequently utilized nanoscale methods include secondary ion mass spectrometry^12^ or sputtered neutral mass spectrometry.^13^ Both of these techniques are destructive and cannot be applied under ambient conditions. Conversely, atom probe tomography can also furnish nanoscale information but is destructive and necessitates time-consuming sample preparation.^14^

Over the past two decades, tip-enhanced Raman spectroscopy (TERS) has emerged as a promising analytical tool^15,16^ for nanoscale molecular characterization across various materials and processes such as two-dimensional (2D) materials,^17−19^ catalysts,^20,21^ polymers,^22,23^ biomaterials,^24−26^ organic photovoltaic devices,^27^ self-assembled monolayers,^28,29^ surface chemical reactions, etc.^30^ TERS employs a metallic scanning probe microscopy tip positioned at the focal point of an excitation laser, which generates an enhanced and confined electromagnetic field (also called near-field) at the tip apex through a combination of the localized surface plasmon resonance and lightning rod effect.^31−33^ This intense and localized near-field at the TERS tip enables the circumvention of conventional Raman microscopy’s diffraction-limited spatial resolution and enhances sensitivity by several orders of magnitude. Leveraging these two effects, enhancement and confinement of the electromagnetic field, TERS can probe nanoscale distribution of molecules. Furthermore, TERS stands out for its unique ability to deliver nanoscale molecular information in a label-free and nondestructive manner in different environments.^34^ Under ultrahigh vacuum and cryogenic conditions, TERS imaging can even visualize Raman vibrational modes within individual molecules with ultrahigh spatial resolution of up to 1.5 Å.^35−37^ However, to the best of our knowledge, TERS has never been applied to the nanoscale chemical analysis of thin film inorganic solar cells.

In this study, we demonstrate for the first time that hyperspectral TERS imaging is a powerful tool to investigate the interfacial regions of absorber (Sb_2_Se_3_) and buffer (CdS) layers in a Sb_2_Se_3_-based thin film solar cell. Hyperspectral TERS imaging, with 10 nm spatial resolution, revealed a blended interface rather than a distinct boundary between the solar cell layers. Moreover, hyperspectral TERS imaging uncovered the penetration of the buffer compound inside the absorber layer. Notably, Raman signals of ZnO from the sputtered window layers were found to be colocalized with the CdS-rich regions within the Sb_2_Se_3_ layer. This work demonstrates the unique potential of hyperspectral TERS imaging for molecular analysis of thin film inorganic solar cells at nanometer length-scales.

## Experimental Methods

### Preparation of Solar Cell Sample

Sb_2_Se_3_-based thin film solar cell investigated in this study was an under-development (noncommercial) lab-scale sample^38−40^ prepared by first depositing a soda-lime commercial glass with a 600–650 nm thick FTO conductive oxide layer followed by the deposition of the following layers on top: (i) 1 μm thick Sb_2_Se_3_ layer deposited by low-temperature pulsed electron beam deposition (PEBS-20 commercial source, supplied by Neocera Inc., MD, USA, at 16 kV discharge voltage and 9 Hz pulse repetition in 3 × 10^–3^ mbar Ar), (ii) 100 nm thick CdS layer deposited by chemical bath deposition at 80 °C, (iii) 150 nm thick layer of undoped ZnO, and (iv) 800 nm thick layer of Al-doped ZnO, both deposited by RF Magnetron Sputtering (Angstrom Sciences, room-temperature, 120 W, 10^–3^ mbar Ar). The solar cell was cut into thin slices for cross-sectional analysis. Before cutting, a second glass was fixed on top using cyanoacrylate glue to protect the solar cell surface. Cutting was performed with a semiconductor-grade diamond annular blade. The obtained slices were fixed on a silicon holder and thinned using P600 and P1000 diamond abrasive papers in a benchtop lapping machine and finally polished using a polishing cloth for semiconductor samples. In order to minimize the risk of sample damage, a well-established standardized procedure from the semiconductor research field was applied for the lapping and polishing of solar cell samples, whereby a sample is thinned by progressive removal of the material. Furthermore, to avoid anisotropic damage to the sample, lapping was performed in a nondirectional manner by continuous rotation of the sample. The intactness of the sample was confirmed by the reproducibility of TERS maps measured in different regions of the solar cell.

### TERS Probe Preparation

To prepare TERS probes, Si atomic force microscopy (AFM) cantilevers were first oxidized in a furnace (Carbolite Gero, UK) at 1000 °C for 23 h to decrease the refractive index of the surface, followed by a UV–ozone (Ossila, UK) cleaning for 1 h. The cleaned AFM cantilevers were placed in a N_2_ glovebox (MBraun, Germany) equipped with a built-in thermal evaporation system and coated with a 150 nm thick layer of Ag (99.95%, Alfa Aesar, USA) at a rate of 0.05 nm/s under 1 × 10^–7^ mbar pressure. To avoid contamination, Ag coated probes were stored in the N_2_ glovebox with less than 0.1 ppm of O_2_ and H_2_O concentration.

### TERS Measurements

The TERS measurements were performed in side-illumination geometry using an integrated system consisting of a Raman spectrometer (LabRam Soleil, HORIBA Scientific, France) combined with AFM (HORIBA Scientific, France). Excitation laser of 532 nm wavelength (λ) was incident on the sample at an angle of 60° with respect to the surface and focused at the TERS probe-apex using a 100×, 0.7 numerical aperture (NA) objective lens. TERS mapping was performed with a laser power of 263 μW at the sample and spectrum acquisition time of 5 s. TERS measurements were performed in “SpecTop mode”, where the AFM feedback switches between tapping and contact modes. During spectral recording, the tip is kept in contact with the sample by using contact mode feedback, whereas the transition from one pixel to the next occurs in tapping mode feedback.

### SEM Measurements

SEM measurements were performed using a Zeiss Auriga Compact Crossbeam equipped with a Gemini electron column for field-emission operation. SEM imaging was conducted using a 5 kV accelerated electron beam and an InLens detector for secondary electrons.

### Data Analysis

TERS spectra were baseline-corrected using polynomial fitting and smoothened using a Savitzky–Golay filter with LabSpec 6 software (HORIBA Scientific, France). The smoothened spectra in Figures 3, 4, S4, and S7 were deconvoluted with Gaussian fitting using OriginPro 2021 (version 9.8.0.200). The TERS maps were generated using the intensity (peak height) of the Raman bands in the baseline-corrected TERS spectra without subtraction of the far-field signal. A description of the data treatment workflow is presented in Figure S1.

**Figure 1:**
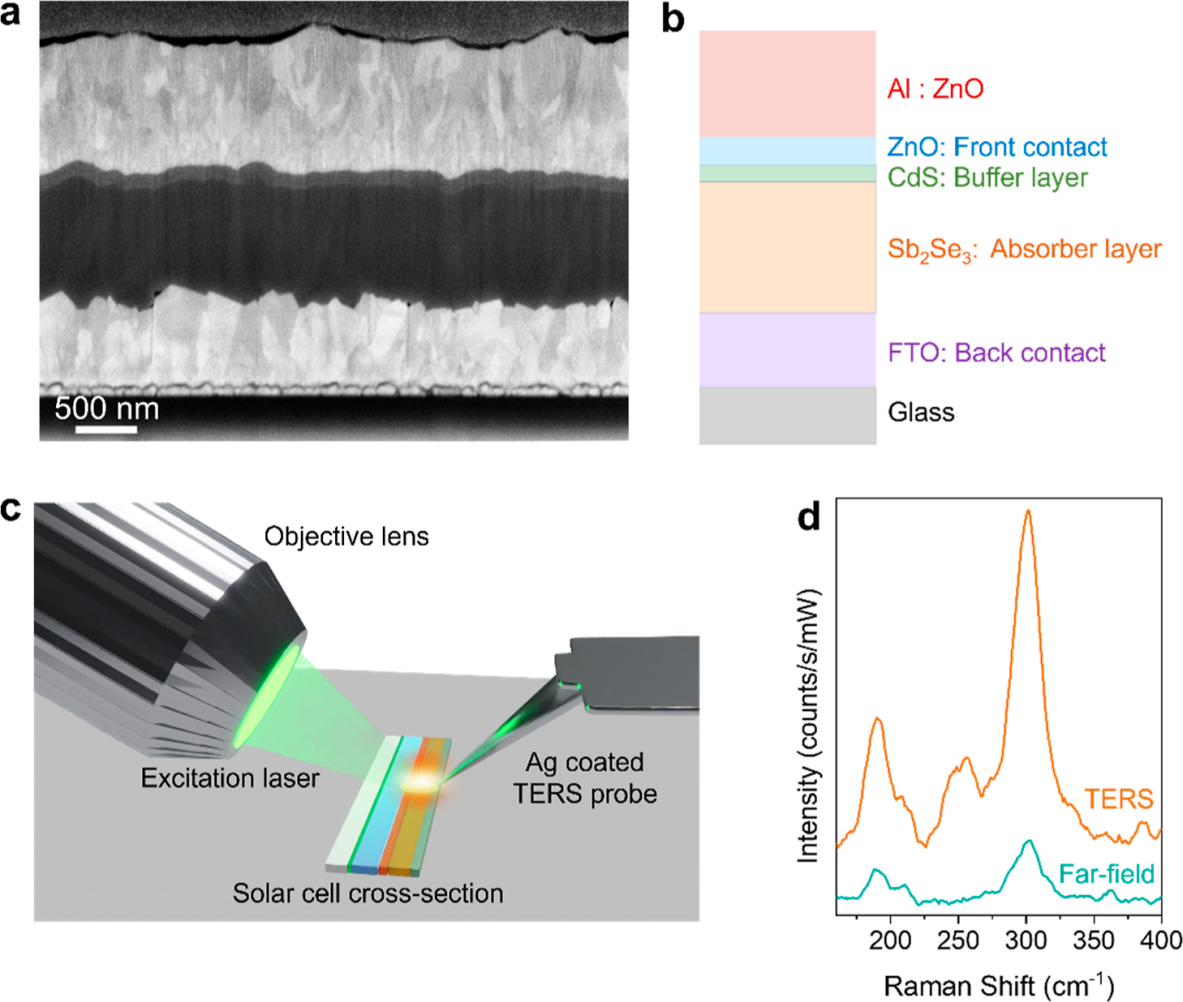
(a) SEM image of the cross section of a Sb_2_Se_3_ thin film solar cell showing its layered structure. (b) Schematic diagram illustrating the multilayer structure of the Sb_2_Se_3_ solar cell. (c) Schematic diagram of the side illumination AFM-based TERS setup used for the interfacial nanoanalysis of the Sb_2_Se_3_ solar cell in this study. (d) Comparison of the far-field Raman (green) and TERS (orange) spectra measured at a similar location on the Sb_2_Se_3_ solar cell. The spectra are normalized to the laser power and integration time.

**Figure 2:**
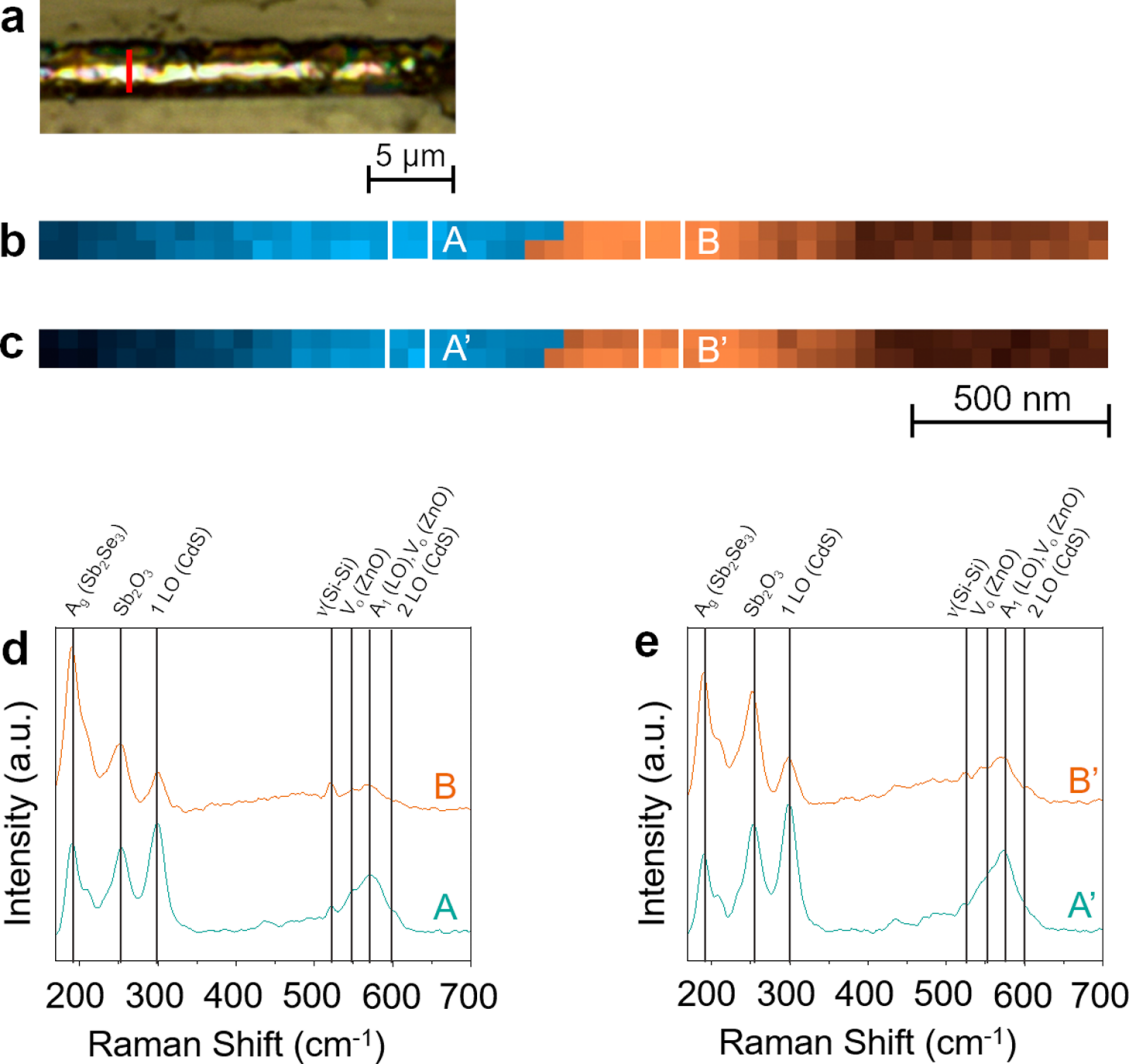
(a) Optical image of a Sb_2_Se_3_ thin film solar cell cross-section. The marked red line indicates the location of the TERS line map. (b) Overlay of the TERS maps constructed using the intensity of Sb_2_Se_3_ (190 cm^–1^, orange), and CdS (300 cm^–1^, blue) Raman signals. Step size: 50 nm. (c) Overlay of the second set of TERS maps recorded consecutively at the same location on the solar cell as the TERS map in Panel b. Average TERS spectra of four pixels measured at the locations marked in (d) Panel b and (e) Panel c.

## Results and Discussion

SEM image of a Sb_2_Se_3_ thin film solar cell cross-section is displayed in Figure 1a alongside a schematic depiction of the corresponding multilayer structure in Figure 1b. An optical image of the sliced Sb_2_Se_3_ solar cell is shown in Figure S2. In our TERS setup, the theoretical size of the diffraction-limited focal spot of the excitation laser is calculated to be 927 nm using the eq (1.22 × λ)/NA, (NA = 0.7 and λ = 532 nm). Since we have a side-illumination geometry, the actual shape of the laser spot on the sample is elliptical and much larger than 927 nm along the major axis. Considering the large size of the excitation laser spot, it is unfeasible to analyze the interface between the individual solar cell layers using conventional Raman spectroscopy due to the lack of nanoscale spatial resolution. Consequently, the interfacial nanoanalysis of the Sb_2_Se_3_ thin film solar cell was performed using TERS as schematically depicted in Figure 1c. We first tested the plasmonic sensitivity of our Ag-coated TERS probes on the solar cell sample. Figure 1d shows a comparison of the far-field Raman and TERS spectra measured at the same location of the sample. Notably, TERS signal intensity is enhanced by a factor of 5.5× compared to the far-field Raman measurement, confirming the high plasmonic sensitivity of TERS probes.

We next tested the reproducibility of the hyperspectral TERS mapping in our experimental setup. Two TERS line maps (step size: 50 nm) were consecutively measured on the same location of the Sb_2_Se_3_ solar cell cross-section (marked in Figure 2a). To visualize the distribution of Sb_2_Se_3_ and CdS, 190 cm^–1^ (A_g_ vibrational mode) and 300 cm^–1^ (1 LO vibrational mode) marker bands were used.^41−43^ Overlay images of the TERS line maps of Sb_2_Se_3_ and CdS TERS signals measured across the solar cell are shown in Figure 2b,c, respectively. The distribution of Sb_2_Se_3_ (orange) and CdS (blue) in the two TERS maps matches very well, confirming the reproducibility of consecutive TERS measurements. Furthermore, average TERS spectra of four pixels measured at the same locations in both TERS maps are presented in Figure 2d,e. TERS spectra of the corresponding locations show the same Raman bands. Interestingly, a relatively higher Sb_2_O_3_ signal at 255 cm^–1^ was observed in the second TERS map due to the laser-promoted oxidation of Sb_2_Se_3_. Analysis of TERS maps generated using the Sb_2_O_3_ signal at 255 cm^–1^ presented in Figure S3 shows 110% increase in the relative intensity of the Sb_2_O_3_ signal, confirming that a longer laser irradiation time promotes the oxidation of the Sb_2_Se_3_ layer. Furthermore, TERS spectra measured with the acquisition times of 5 and 10 s (Figure S3d) show 64% increase in the *I*_Sb2O3_/*I*_Sb2Se3_ ratio further signifying that a longer laser irradiation promotes Sb_2_Se_3_ oxidation. These observations are in line with the previous report on the oxidation of Sb_2_Se_3_-based thin film solar cell by Spaggiari et al.^40^

**Figure 3:**
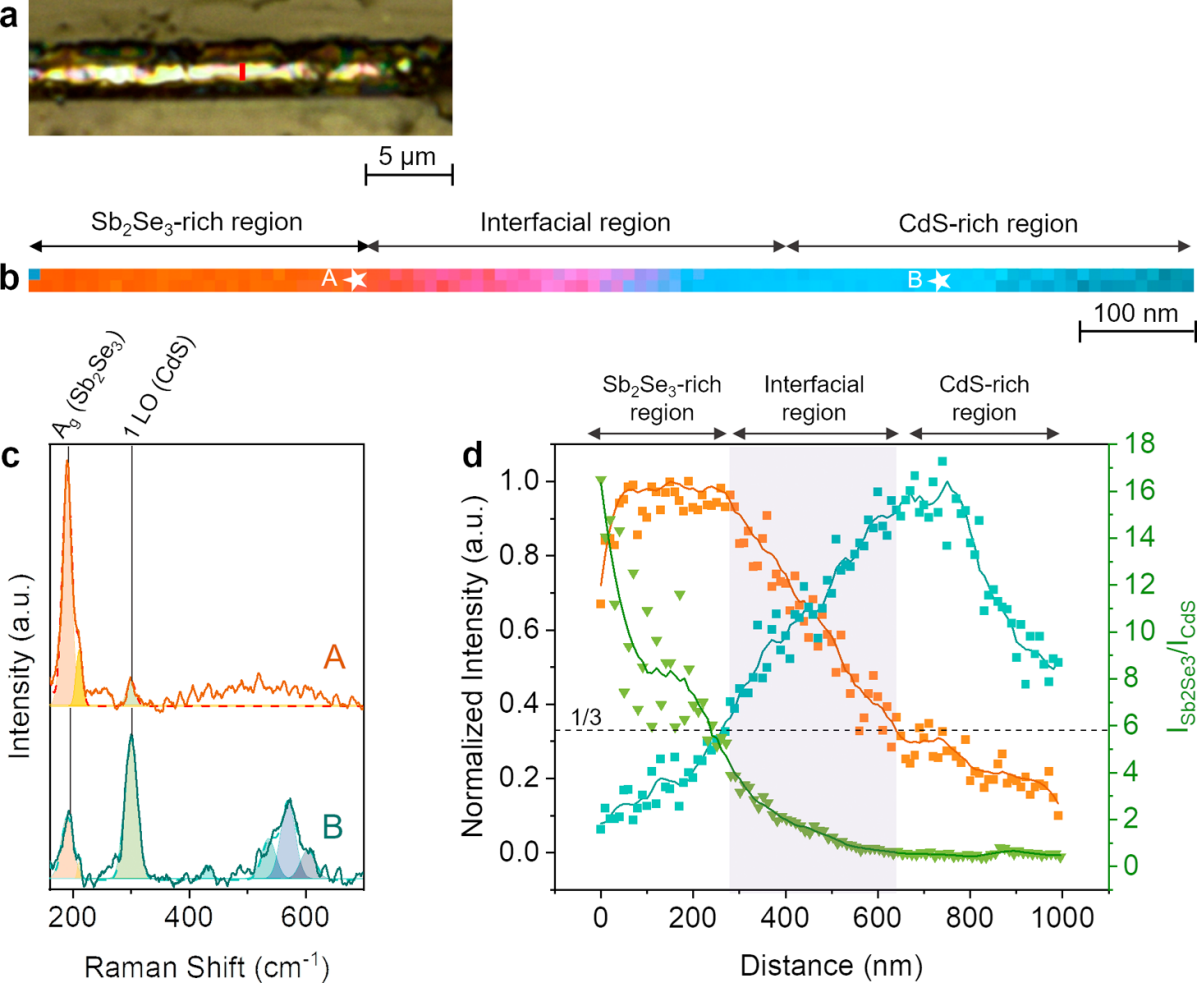
(a) Optical image of a Sb_2_Se_3_ thin film solar cell cross-section. The marked red line indicates the location of the TERS line map. (b) Overlay of the TERS maps constructed using the intensity of Sb_2_Se_3_ (190 cm^–1^, orange) and CdS (300 cm^–1^, blue) Raman signals. Step size: 10 nm. (c) TERS spectra measured at the locations marked in Panel b. (d) Plot of the CdS (300 cm^–1^, blue) and Sb_2_Se_3_ (190 cm^–1^, orange) TERS signals and their ratio (*I*_Sb2Se3_/*I*_CdS_) measured from right to left in Panel b. The interfacial region is highlighted by a purple band.

For a more detailed analysis of the Sb_2_Se_3_/CdS interface, we performed TERS measurements with a much smaller step size of 10 nm. An optical image of the Sb_2_Se_3_ solar cell slice is shown in Figure 3a and overlay of the TERS line maps of Sb_2_Se_3_ (orange) and CdS (blue) signals measured across the solar cell (marked in Figure 3a) is shown in Figure 3b. TERS spectra measured at locations A and B (marked in Figure 3b) are presented in Figure 3c. At location A, 190 cm^–1^ is the most intense peak confirming that it is a Sb_2_Se_3_-rich region. On the other hand, at location B, the 300 cm^–1^ peak is the most intense, confirming the existence of a CdS-rich region. A weak Sb_2_Se_3_ signal is observed in the CdS-rich layer and vice versa, which likely arises from the far-field. Besides the 190 and 300 cm^–1^ bands, TERS spectra also display additional Raman bands that are labeled in Figure S4 and assigned in Table 1. The high-resolution hyperspectral TERS imaging in Figure 3b demonstrates that the Sb_2_Se_3_-rich (orange) and CdS-rich (blue) layers of the solar cell can be successfully distinguished at the nanometer length-scale.

**Table 1:** Assignment of the Raman Peaks Observed in the TERS Spectra of the Sb_2_Se_3_-Based Thin Film Solar Cell

Raman peak (cm^–1^)	vibrations	compound	reference
190	A_g_	Sb_2_Se_3_	(41,42)
210	A_g_	Sb_2_Se_3_	(41,42)
255	–	Sb_2_O_3_	(40)
300	1 LO	CdS	(43)
433	E_2_ LO	ZnO	(44)
549	V_o_	ZnO	(44)
574	A_1_ (LO), V_o_	ZnO	(44)
600	2LO	CdS	(43)

Notably, the Sb_2_Se_3_ and CdS layers of the solar cell lack a distinct boundary; instead, a transition region of interpenetrating compounds is observed, as depicted in the TERS intensity profile in Figure 3d. We term this area as the “interfacial region” and define it as the region where Raman signals of both Sb_2_Se_3_ and CdS exceed 1/3rd of the highest signal in their respective layers. Figure S5 showcases two additional TERS line maps measured in different regions across the solar cell and their corresponding intensity profiles, which exhibit similar behavior. Utilizing the defined criterion, we estimate the length of the interfacial region between the Sb_2_Se_3_ and CdS layers from the intensity profiles in Figures 3d and S5 to be 295 ± 70 nm. Interestingly, this measurement is significantly larger than the ca. 60 nm interfacial length previously reported for Cu(In,Ga)Se_2_-based solar cells, where the buffer layer was also deposited using the chemical bath deposition method.^7^ The increased length of the interfacial region in our solar cell can be attributed to the high surface roughness of the Sb_2_Se_3_ layer as shown in the SEM images of pristine Sb_2_Se_3_ in Figure S6, where needle-like protrusions are observed at the surface. TERS measurements in areas where the CdS layer covers these needle-like protrusions will yield a thicker interfacial value. Notably, a similar size of the interfacial region was consistently found in several line maps (Figure S5), signifying that the interfacial region is significantly modified by the Sb_2_Se_3_-layer roughness.

**Figure 4:**
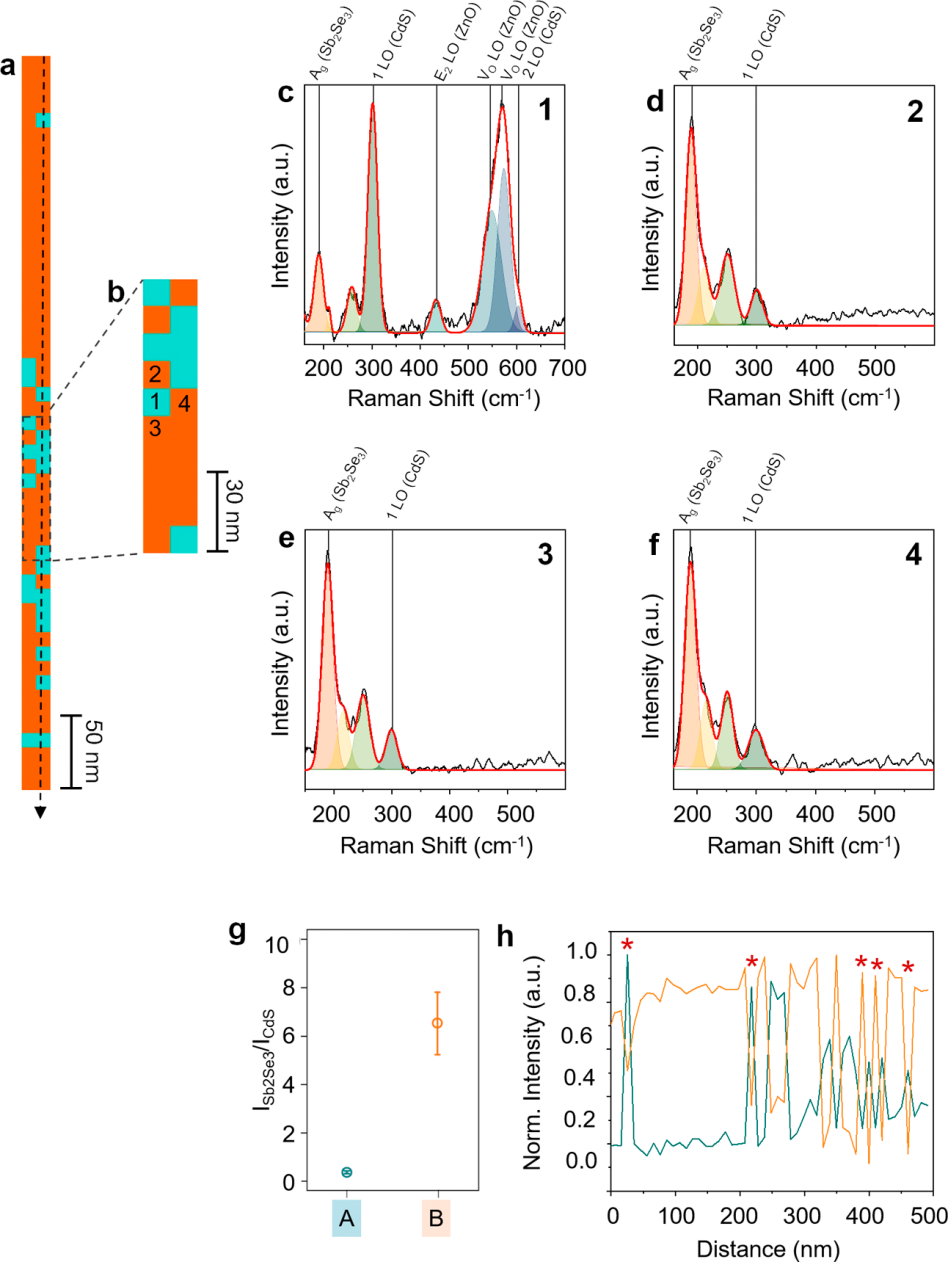
(a) Overlay of the TERS maps of the Sb_2_Se_3_ (190 cm^–1^, orange) and CdS (300 cm^–1^, blue) Raman signals. Step size: 10 nm. (b) Zoomed-in image of a portion of the TERS map highlighted by a dashed rectangle in Panel a. (c) TERS spectrum measured at the pixel marked as 1 in Panel b. Prominent peaks in the spectrum are fitted using Gaussian curves. Strong CdS (300 cm^–1^) and ZnO (433, 549, and 574 cm^–1^) signals are observed at this position. (d–f) TERS spectra measured at the pixels marked as 2–4 in Panel b fitted using Gaussian curves. A strong Sb_2_Se_3_ signal (190 cm^–1^) is observed in these spectra. (g) Intensity ratio (*I*_Sb2Se3_/*I*_CdS_) plot of the Raman signals in the CdS-rich region (blue) and Sb_2_Se_3_-rich region (orange) shown in Panel a. (h) CdS (300 cm^–1^, blue) and Sb_2_Se_3_ (190 cm^–1^, orange) TERS signal profiles measured along the line (top to bottom) marked in Panel a.

Hyperspectral nanoscale TERS imaging also identified the nanoscale penetration of CdS inside the Sb_2_Se_3_ layer. An overlay of the TERS line maps of CdS (blue) and Sb_2_Se_3_ (orange) signals measured inside the Sb_2_Se_3_ layer is displayed in Figure 4a where several nanoscopic CdS-rich regions are observed. To visualize this more clearly, a zoomed-in image of a portion of the measured region is shown in Figure 4b. TERS spectra recorded at the pixels marked as 1–4 are shown in Figure 4c–f, respectively. The TERS spectrum at pixel 1 exhibits a dominant CdS Raman signal, whereas the TERS spectra from pixels 2–4 show dominance of the Sb_2_Se_3_ Raman signal. Four additional TERS spectra measured at the blue pixels in Figure 4a are shown in Figure S7 confirming the dominance of CdS at these locations. Furthermore, the average intensity ratio (*I*_Sb2Se3_/*I*_CdS_) of the Raman signals measured in the orange and blue regions of Figure 4a is found to be 0.6 and 6.5, respectively (Figure 4g). This correlates very well with the *I*_Sb2Se3_/*I*_CdS_ ratio of the Sb_2_Se_3_-rich and CdS-rich areas observed in Figure 3d. These results unequivocally confirm the presence of nanoscale CdS deposits inside the Sb_2_Se_3_ layer.

This finding is in agreement with the previous report of the CuInSe_2_-based solar cells by Cojocaru-Mirédin et al., where open pores in the CuInSe_2_ absorber layer were filled by CdS.^14^ The open pores have been postulated to form via etching of the absorber surface by the reagents used during the chemical bath deposition process. Notably, the occupation of open pores in the absorber layer by CdS can improve the defect passivation effect and facilitate charge carrier transport, thereby enhancing the power conversion efficiency.

In the Sb_2_Se_3_ solar cell, ZnO is the n-type component of the p–n junction situated on top of the CdS buffer layer as depicted in Figure 1b. In addition to CdS, the presence of ZnO inside the Sb_2_Se_3_ layer was also revealed by hyperspectral TERS imaging. The TERS spectrum displayed in Figure 4c exhibits 433 cm^–1^ peak, which is assigned to ZnO.^44,45^ Furthermore, the 500–600 cm^–1^ region exhibits several high intensity peaks (deconvoluted using Gaussian fitting), where the 549 cm^–1^ peak is assigned to the vibrational modes associated with the oxygen vacancies (V_o_) in ZnO, while the 574 cm^–1^ peak is assigned to a combination of the A_1_ (LO) eigenmode of ZnO and the V_o_ mode of oxygen vacancies.^44^ Therefore, the 433, 549, and 574 cm^–1^ peaks confirm the presence of ZnO inside the Sb_2_Se_3_ layer, which interestingly is colocalized with the nanoscale CdS deposits (Figures 4c and S7). To the best of our knowledge, this is the first report of the penetration of ZnO down to the absorber layer. This observation is important because these heterojunctions, where the n-type ZnO layer is separated from the p-type Sb_2_Se_3_ layer by the CdS buffer layer, have been proven to work very efficiently. Therefore, ZnO is not intended to reach the Sb_2_Se_3_ absorber layer. These results indicate that the deposition of ZnO on the top of CdS by radio frequency magnetron sputtering, which is a high-energy technique, can induce the excessive (and undesirable) penetration of ZnO.

To demonstrate the high spatial resolution of TERS mapping, intensity profiles of the Sb_2_Se_3_ and CdS Raman signals along the line (top to bottom) marked in Figure 4a are plotted in Figure 4h. As discussed earlier, a negative correlation is observed between the Sb_2_Se_3_ and CdS signals. Notably, at the locations marked with stars in Figure 4h, an abrupt intensity change from the Sb_2_Se_3_ to CdS signals is observed within 10 nm (one pixel). If we define the spatial resolution of an image to be the smallest sample feature distinguished, the lateral resolution of the TERS map in Figure 4a is estimated to be 10 nm.

Note that the TERS maps in Figures 2–4 were constructed without subtracting the far-field Raman signal from the TERS spectra due to the limitation of our TERS setup, where it was not possible to record a far-field Raman map at exactly the same position where the TERS map was recorded. However, as shown in Figure 1d, the TERS signal is 5.5 times stronger than the far-field signal, which signifies a strong plasmonic enhancement of Raman signals in the near-field. Furthermore, the excitation laser in our setup has a focal spot of 927 nm, which is significantly bigger than the 10–50 nm step size used during TERS mapping. Therefore, the relatively minor contribution of the far-field to the TERS signal is not expected to substantially alter the key conclusions of this study.

## Conclusions

In this work, hyperspectral TERS mapping was applied to investigate a Sb_2_Se_3_ solar cell with a high spatial resolution of 10 nm. Our findings unequivocally demonstrate that the interface between the absorber and the buffer layer constitutes a region of mixed chemical composition: TERS analysis revealed an intermixing rather than a sharp demarcation between the CdS and Sb_2_Se_3_ layers. Notably, this interface, containing both Sb_2_Se_3_ and CdS compounds, exhibited an unexpectedly large thickness of 295 ± 70 nm, a phenomenon that is partially attributable to the high roughness of the Sb_2_Se_3_ layer and to the chemical bath process used to deposit CdS. A further confirmation of the latter hypothesis came from the compelling evidence of nanoscale CdS deposits inside the Sb_2_Se_3_ layer provided by the TERS measurements. This likely results from the filling of pores in the absorber surface formed during the chemical bath deposition process.^14^ Additionally, Raman peaks at 433, 549, and 574 cm^–1^ also indicated the presence of ZnO colocalized with the CdS deposits inside the Sb_2_Se_3_ layer, which is the first experimental report of an unintentional ZnO penetration down to the Sb_2_Se_3_-rich region. Notably, although the specific results of this study, especially concerning sample-specific attributes and preparation methods, may not be generalizable to all Sb_2_Se_3_-based inorganic solar cells, they demonstrate that TERS is an underutilized yet highly potent tool for nanoscale interfacial analysis in thin film solar cells. TERS can provide a fresh nanoscale perspective via characterization of chemical compositions, phases, defects, strain/stress, etc. in thin film heterostructures and position itself as a powerful complementary analytical tool alongside conventional methods.

## Data Availability

The original data used in this publication are made available in a curated data archive at ETH Zurich (https://www.research-collection.ethz.ch) under the DOI 10.3929/ethz-b-000640017.

## Supporting Information Available

Example of data treatment, optical images of the solar cell sample, TERS map of the oxidation Raman marker band, representative TERS spectrum of the Sb_2_Se_3_ solar cell, additional TERS imaging data, SEM images of the pristine Sb_2_Se_3_ film, and additional TERS spectra from nanoscale CdS deposits in the Sb_2_Se_3_ layer PDF
