# Supplementary material for: Nanoscale Chemical Analysis of Thin Film Solar Cell Interfaces Using Tip-Enhanced Raman Spectroscopy
Source: ACS Appl Mater Interfaces. 2024 Mar 18;16(12):14704–11. doi: 10.1021/acsami.3c17115 (PMC10982994; doi:10.1021/acsami.3c17115)
Supplement: Supplementary file 1 — am3c17115_si_001.pdf [file am3c17115_si_001.pdf]

Supporting Information

Nanoscale Chemical Analysis of Thin Film Solar Cell Interfaces using Tip-Enhanced Raman Spectroscopy

Siiri Bienz,[(a)§] Giulia Spaggiari,[(b,c)§] Davide Calestani,[(c)] Giovanna Trevisi,[(c)] Danilo Bersani,[(b)] Renato Zenobi,[(a)*] and Naresh Kumar[(a)*]*

(a) Department of Chemistry and Applied Biosciences, ETH Zurich, Vladimir-Prelog-Weg 1–5/10, 8093 Zurich, Switzerland

(b) Department of Mathematical, Physical and Computer Sciences, University of Parma, Parco Area delle Scienze 7/A, I-43124 Parma, Italy

(c) Institute of Materials for Electronics and Magnetism, National Research Council, Parco Area delle Scienze 37/A, I-43124 Parma, Italy

*Corresponding authors: danilo.bersani@unipr.it, zenobi@org.chem.ethz.ch, naresh.kumar@org.chem.ethz.ch

[§]S.B. and G.S. contributed equally.

Table of contents:

Figure S1. Data treatment workflow.

Figure S2. Optical image of the sandwiched thin film solar cell sample.

Figure S3. Analysis of the TERS maps of Sb_2O_3 Raman signal

Figure S4. Raman bands observed in a representative TERS spectrum of the Sb_2Se_3 solar cell.

Figure S5. Two additional overlay images of the TERS line maps recorded in the interfacial region.

Figure S6. SEM image of a pristine Sb_2Se_3 film fabricated by low temperature pulse electron deposition.

Figure S7. Additional TERS spectra from the CdS-rich region.

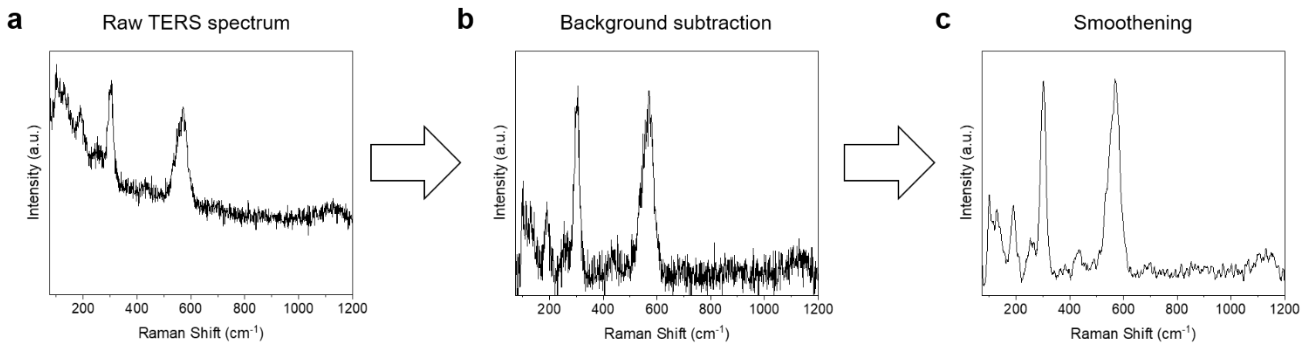

Figure S1. Data treatment workflow. (a) A representative raw TERS spectrum. Acquisition time: 5 s. Laser power at the sample: 263 μW. (b) TERS spectrum was first baseline subtracted by fitting the spectral background with a curve of polynomial order 7. (c) Finally, the TERS spectrum was smoothened using the Savitzky-Golay filter with window length of 15 points and polynomial order of 3.

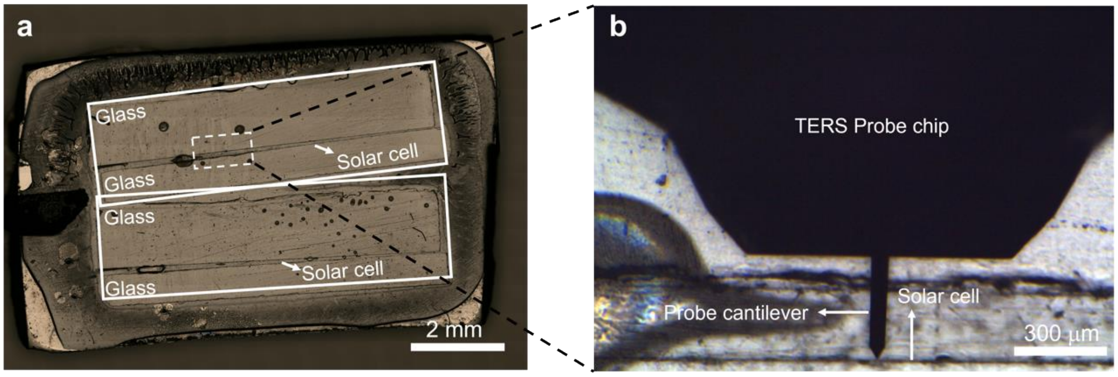

Figure S2. (a) Optical image of the solar cell sample recorded with a 5× objective lens. The solar cell cross-section is sandwiched between two glass slides and is visible as a thin line between two glasses. (b) Optical image of the TERS tip aligned with the solar cell sample recorded with a 10× objective lens. AFM cantilever is visible in black and the solar cell is visible underneath.

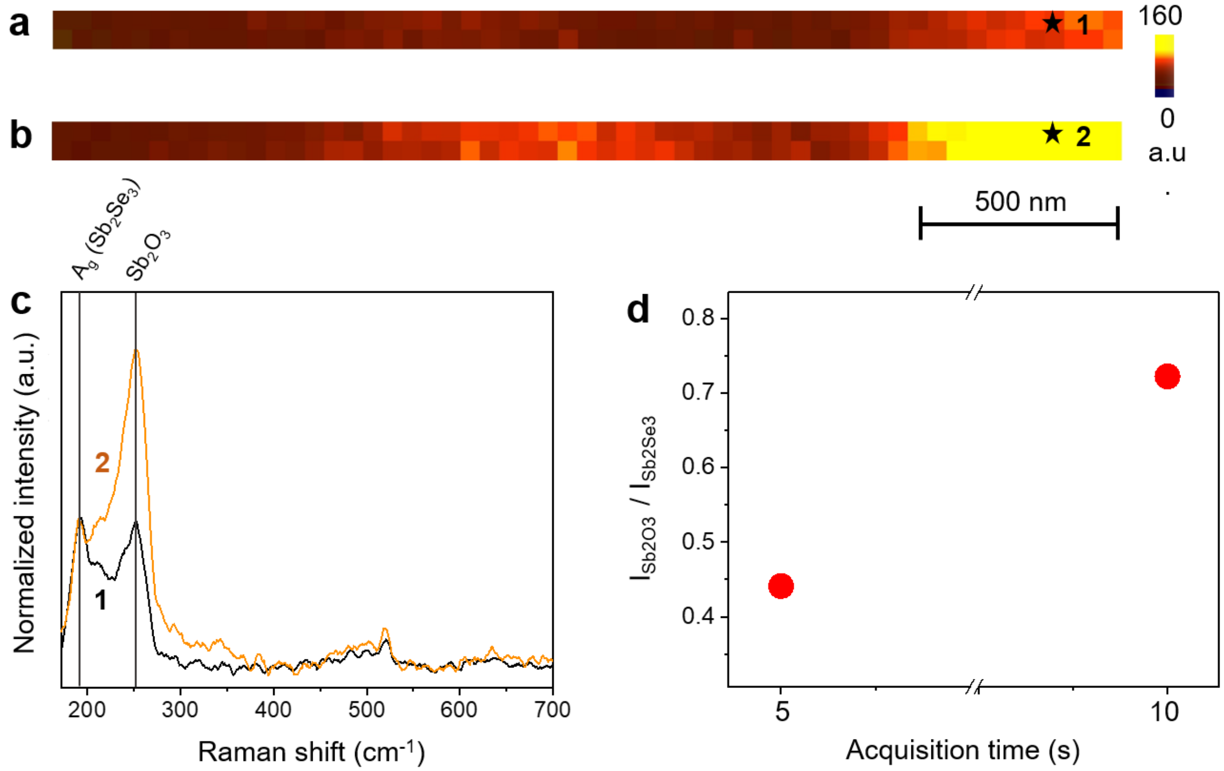

Figure S3. (a) TERS map constructed using the Sb_2O_3 signal at 255 cm^{-1}. Step size: 50 nm. (b) A second TERS map of the Sb_2O_3 signal recorded consecutively at the same location on the solar cell as Panel a. The TERS maps in Panels a and b are constructed from the same hyperspectral TERS measurement as in Figures 2b and 2c, respectively. (c) TERS spectra measured at the locations marked in Panels a and b. Both spectra are normalized to the Sb_2Se_3 signal at 190 cm^{-1}. A 110 % higher Sb_2O_3 signal (255 cm^{-1}) is observed in the second TERS map indicating that a longer laser irradiation time promotes oxidation of the Sb_2Se_3 layer. (d) Comparison of the I_{Sb2O3}/I_{Sb2Se3} ratio in the TERS spectra measured with the acquisition times of 5 s and 10 s. By doubling the spectrum acquisition time, I_{Sb2O3}/I_{Sb2Se3} ratio increases by 64 %, which further signifies that a longer laser irradiation promotes Sb_2Se_3 oxidation. These observations are in line with the previous report on the oxidation of Sb_2Se_3-based thin film solar cell by Spaggiari *et al*.[1]

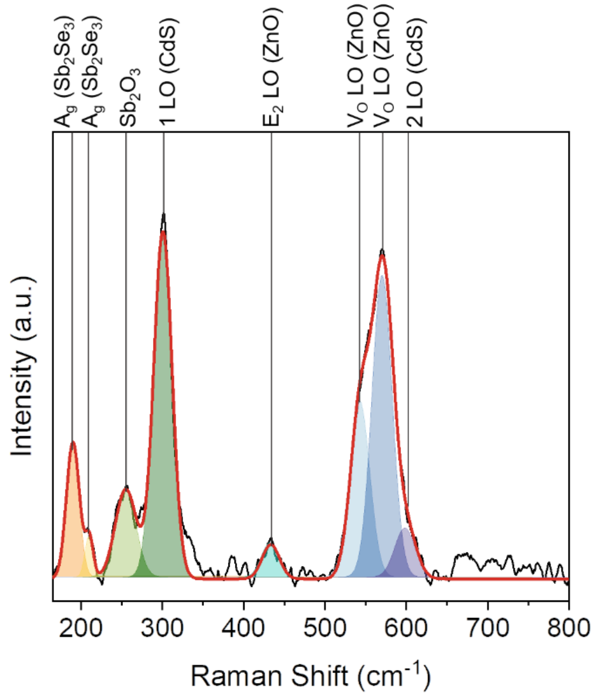

Figure S4. Raman bands labelled in a representative TERS spectrum of the Sb_2Se_3 solar cell measured in a CdS-rich region.

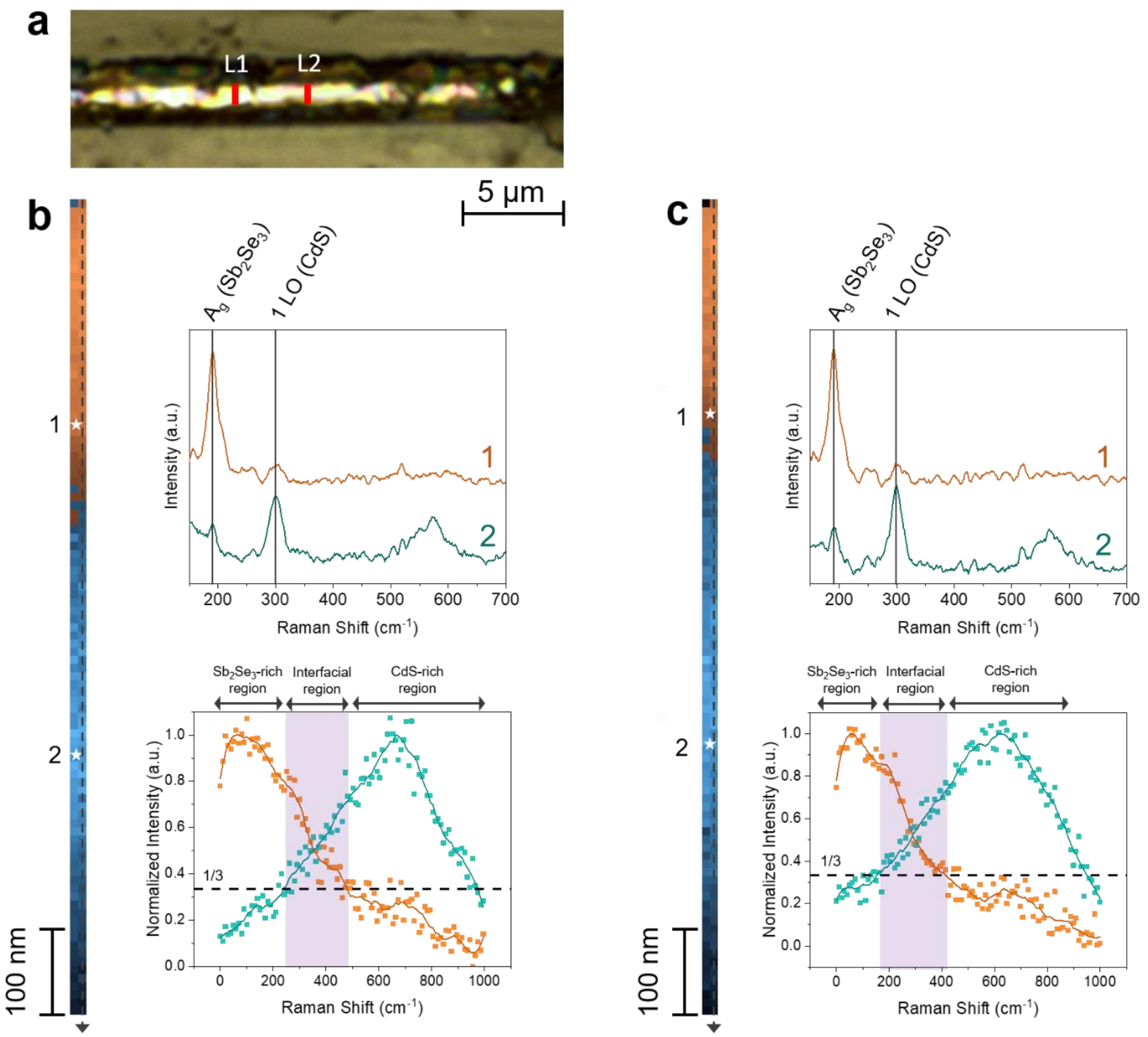

Figure S5. (a) Optical image of the Sb_2Se_3 solar cell cross-section. (b, c) Two additional overlay images of the high-resolution (step size: 10 nm) TERS line maps of CdS (blue) and Sb_2Se_3 (orange) measured along lines L1 and L2 in Panel a and their corresponding intensity profiles. Spectra measured at locations 1 and 2 of the TERS maps confirm the abundance of Sb_2Se_3 and CdS, respectively. A gradual transition rather than a sharp boundary is observed at the Sb_2Se_3/CdS interface. The interfacial region is highlighted with a purple band.

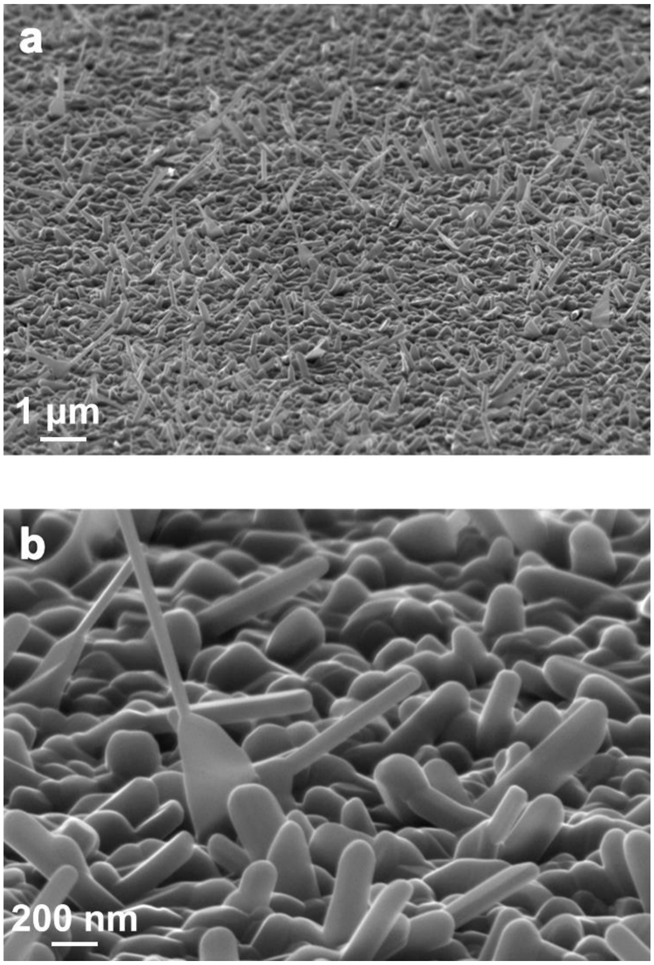

Figure S6. (a) SEM image of a pristine Sb_2Se_3 film fabricated by low temperature pulse electron deposition. The Sb_2Se_3 film displays a very rough surface covered with needle-like protrusions. (b) A zoomed-in SEM image of the Sb_2Se_3 surface. The sharp needle-like surface protrusions can be up to 1 μm in length.

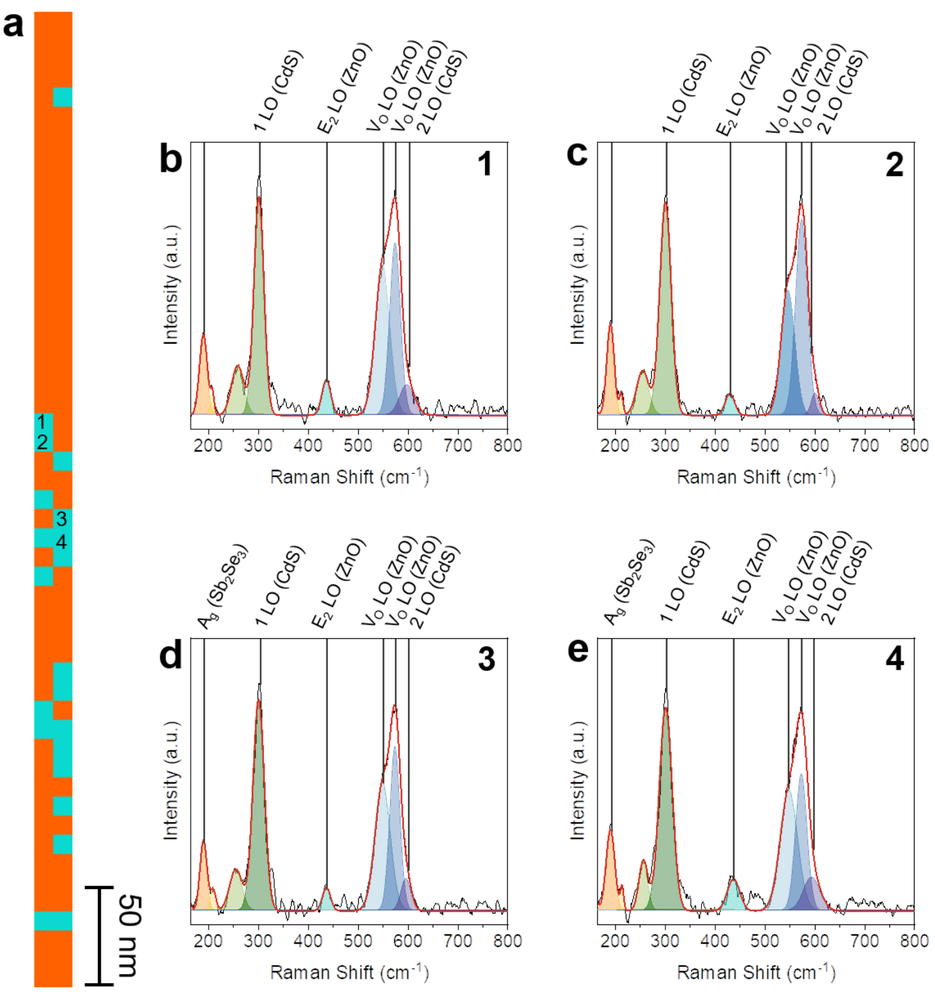

Figure S7. (a) Overlay of the TERS images of Sb_2Se_3 (190 cm^{-1}, orange) and CdS (300 cm^{-1}, blue) Raman marker bands shown in Figure 3a. (b-e) TERS spectra measured at the CdS-rich regions marked as 1-4 in Panel a. Prominent Raman peaks in the TERS spectra are fitted using Gaussian curves. Clear signals of CdS (300 cm^{-1}) and ZnO (433 cm^{-1}, 549 cm^{-1} and 574 cm^{-1}) are observed at all these locations.

Supplementary reference

1. Spaggiari, G.; Pattini, F.; Bersani, D.; Calestani, D.; De Iacovo, A.; Gilioli, E.; Mezzadri, F.; Sala, A.; Trevisi, G.; Rampino, S., Growth and structural characterization of Sb2Se3 solar cells with vertical Sb4Se6 ribbon alignment by RF magnetron sputtering. *J. Phys. D: Appl. Phys.* **2021,** *54* (38), 1-11.
